# American Medical Association

**Published:** 1861-05

**Authors:** 


### EDITORIAL AND MISCELLANEOUS.

### American Medical Association.

It is a matter of some importance, to those in the South who intend being present at the next session of this body in Chicago, that information be given as to the means of protection necessary for *seceders*, during its deliberations.

Will our cotemporary of the Nashville Journal of Medicine and Surgery, in another non-sectional flight above "every line and landmark drawn by the political diplomatist," tell us how he proposes to avoid annoyance from abolition mobs while in Chicago, short of favoring Lincoln's government? There *may* be some who desire to go, in the enjoyment of cherished principles connected with our new Confederacy; and while the Editor of the above Journal "hopes to see a large meeting at Chicago in June next," he should inform the profession that something more than the "unsullied habiliments" of the physician has recently been found necessary to protect Southrons from the assaults of abolition fanatics in Northern cities. In the present state of society North, our opinion is that the "habiliments" of *war*, for Southern men going North with Southern principles, would be an appropriate panoply. And while we think, from information we have had, that the Editor and his *confreres* in the college may be able to furnish a full corps of delegates whose political sentiments will not be objected to at Chicago, we know of many Institutions not thus situated.

